# Application of Raman Spectroscopy to Ancient Materials: Models and Results from Archaeometric Analyses

**DOI:** 10.3390/ma13112456

**Published:** 2020-05-28

**Authors:** Daniele Chiriu, Francesca Assunta Pisu, Pier Carlo Ricci, Carlo Maria Carbonaro

**Affiliations:** Department of Physics, University of Cagliari—Cittadella Universitaria, 09042 Monserrato, Italy; francescaassunta.pisu@dsf.unica.it (F.A.P.); carlo.ricci@dsf.unica.it (P.C.R.); cm.carbonaro@dsf.unica.it (C.M.C.)

**Keywords:** Raman spectroscopy, mathematical model, cultural heritage

## Abstract

Numerous experimental techniques of analysis find applications in many branches of the archaeometry. Among them, Raman spectroscopy carved out a niche in the field of diagnostic and conservation of cultural heritage. The exceptional ability to predict and discover the structural properties of materials set for Raman spectroscopy, an exclusive role among the analytic techniques, is further boosted when it is coupled with mathematical or statistical models able to deepen the studied phenomena. In this work, we present a review of recent studies where pairing Raman spectroscopy and mathematical models allowed achieving important results in the case of potteries, porcelains, ancient and modern paper, ancient jewelry, and pigment degradation. The potentialities of this approach are evidenced and analyzed in detail.

## 1. Introduction

In the last decades, Raman spectroscopy gained a key role in the field of cultural heritage because of its exceptional ability to predict and discover the structural properties of materials [1,2,3,4]. The peculiarity of this technique consists in its non-destructive character and its high sensitivity, also for portable instrumentations, a characteristic highly desirable for field test of precious ancient relics [5,6,7,8,9]. In addition, it can be considered a thorough technique for compositional analysis: it is able to study not only inorganic materials (e.g., crystalline phases and their transitions), but also organic materials or functional groups, like OH hydroxy ions related to hydration conditions of a specific compound [10,11]. For what concerns inorganic materials this technique is complementary to other reference techniques like X-Ray Diffraction (XRD) and X-Ray Fluorescence (XRF) the former being an accurate but destructive laboratory technique, the latter a non-destructive one, also sharing with Raman spectroscopy high sensitivity and portability characteristics. However, Raman spectroscopy surpasses the limit of mentioned techniques with regards to the detection of light elements (in particular, present portable XRF instrumentations can only detect elements with atomic number Z > 11), which barely allows for discrimination of an organic compound [12,13].

Therefore, the research, especially in the field of the cultural heritage, seeks for continuous steps forward in Raman spectroscopy to improve the following characteristics.

- Higher sensitivity: In order to provide a ppm detection threshold, for example, Surface Enhanced Raman Scattering (SERS) was developed [14,15].
- Portability: Very light devices have been produced in order to have good facility of handling and transport [16,17].
- Spatial sampling: Automated imaging tools are developed in order to have a good representativeness of the sampled area where compositional analyses are mixed with a surface mapping [18,19].
- Sub-layers analysis: A recent variant of Raman spectroscopy, Spatially Offset Raman Spectroscopy (SORS), was implemented to study the stratigraphy of layered relics [20,21].

**Figure 1:**
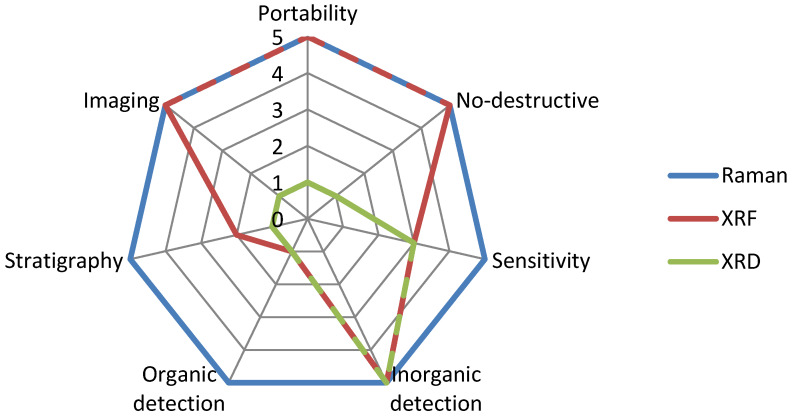
Comparison of some relevant characteristics associated to three different experimental techniques: Raman (blue), X-ray fluorescence (XRF) (red), and X-ray diffraction (XRD) (green).

To compare Raman, XRF, and XRD techniques we merely reported some relevant characteristics on a 1 to 5 scale in Figure 1. As evidenced by the graph, albeit the complementarity of these techniques, Raman spectroscopy shows numerous advantages with respect to the other two techniques. 

The ability of Raman spectroscopy as an analytical tool to individuate structural and compositional properties of materials is further increased to define the temporal and geographical trajectories of analyzed relics when Raman spectroscopy is coupled to some specific mathematical model or statistical analysis. Clearly, particular attention deserves to be paid to the accuracy of the model, which reflects the quality of the analysis and contributes to estimate the reliability of the model.

Once a specific physical observable is individuated as relevant to define the history of an artifact under investigation, such as the presence of some vibrational features or the ratios (of intensities or areas) of such fingerprints, the band shifts, or the band width (Full Width Half Maximum (FWHM)), one can investigate how this observable is related to external parameters, such as temperature *T*, light energy *E (hν)*, relative humidity *RH*, pH, etc. Those parameters are typically related to the technological knowledge of the historic period and the environment characteristics, the one where the artifact was realized and then conserved, affecting the aging of the manufact. Thus, by studying these observables one can gather information about the technique of the art maker, the raw materials used and the production process, pursue dating targets or conservation state purposes, or determine possible agents responsible for or able to degrade the investigated artifacts. 

As for aging studies, the connection of Raman observables and aging parameters can lead to different models, the most common ones being represented by kinetic (first- or second-order), Arrhenius, and logistic models, whose formulation allows singling out the effect of a specific parameter across the time, eventually with the help of experimental archeology samples [22]. Sometimes the exploitation of computational chemistry simulations of the material structure under investigation can help to identify the mentioned aging or degradation characteristic parameters, when specific information cannot be retrieved from experimental data. On the other hand, when focusing on geographical trajectories and specific provenance of investigated materials, one can divide some selected observables in different classes, allowing the use of multivariate (MV) models [23]. Raman technique is suitable to provide classes of observables which can be used, for example, to activate a Principal Component Analysis (PCA) [24]. 

In this review, we present some recent important applications of Raman spectroscopy to show that when Raman investigation is paired to mathematical models or a multivariate approach this synergic strategy allows to deepen the study of cultural heritage relics and to define specific temporal and geographical trajectories of the manufacts across history. To show the power of this strategic approach, we report the application of this technique to different relevant materials in cultural heritage investigations, like ceramics, ancient paper, pigments, gemstones, and some other illustrative case where the combined use of Raman spectroscopy and a specific predictive model was and can be successfully exploited.

## 2. Results Overview

The aim of this review is to promote Raman spectroscopy not only as a sensitive, nondestructive, and in-field portable analytical technique of success for cultural heritage, but to spread its application as a powerful predicting tool when combined to mathematical and statistical models. In the following, we report some interesting illustrative case where this synergic approach was applied. The rationale for the selected cases is twofold: first, to show that the strategy is successful in different areas of the cultural heritage characterized by different analyzed materials, from ceramics to pigments, and second to evidence that the joint Raman analysis and modeling can support cultural heritage researchers in tracing the history of the analyzed relics. We will see that if the appropriate observable is selected this approach, combined with history context knowledge, can help to assess the age of a relic, the artwork technology exploited to realize the manufact or possible commercial routes in the examined historical period, as for the ceramics (Section 2.1). The strategy is also fruitful when facing dating issue for authenticity or preservation purposes, as for books and paintings (Section 2.2), or when we are concerned about the geographical provenance of some jewel and we would like to correlate it to a specific historical period (Section 2.3).

### 2.1. Ceramic Materials

**Figure 2:**
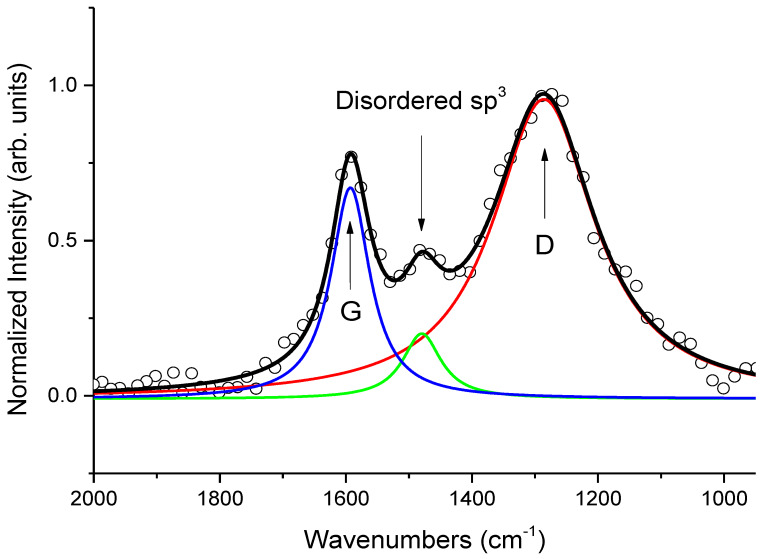
Simulated D (red), G (blue), and disordered sp^3^ (green) bands in Amorphous Carbon detected in clay samples from Khirbat Iskandar. Ratio between areas and intensities of D and G bands were used to determine production parameters of clay. (Reproduced from work in [25] with the permission of Elsevier, copyright 2018.).

A first example of combination between Raman and mathematical model is reported in [25] (Figure 2). In this paper, we performed a near-infrared micro-Raman investigation (with a portable instrumentation with excitation wavelength at 1064 nm) on Early Bronze IV pottery from Khirbat Iskandar (in the old Canaan land, Dhiban, Jordan). For all the experimental conditions we refer to the cited work. 

When the target of the investigation is a complete study of pottery composition, the latter can be obtained by devoting particular attention to three distinctive phases of the manufact production: the primary phase, involving natural unprocessed materials or other intentionally added by potters (as the temper, for example); the secondary phase, which is related to the firing processes and the realization of new compounds; and, to conclude, the third phase, involving the production of new constituents out of the artifact exploitation or related to the burial context [26]. Through the study of secondary phase, in addition to the redox state of the firing environment, the highest firing temperature can be estimated by studying characteristic parameters which depend on the process (e.g., hematite/magnetite ratio and production or dissolution of components during the firing). 

In the reviewed paper, beside the other findings, among which it is important to cite the presence of Olivine and anatase for geographical tracing purposes, we detected the presence of amorphous Carbon, identified as temper in the clay. The Raman spectrum of the latter was investigated in detail to estimate relevant characteristics of the manufacturing process, such as maximum firing temperature and furnace permanence time. To analyze the data, we performed a deconvolution of the experimental curve by means of Lorentian bands, and we extracted the well-known D and G bands of graphite-like compounds. 

Assuming the ratio between the areas of the two bands (*A_D_*/*A_G_*) and the peak positions as the starting point, we estimated the sample firing temperature to be 700°C through the ratio between the intensity of the two simulated *I_D_*/*I_G_* bands and their correlated width (FWHM). In addition, by assuming the model of Ferrari et al. [27] applied to the band at 1480 cm^−1^ (the green one in the figure, related to disordered sp^3^ structures), we evidenced that clustering of sp^2^ hybridization of graphite-like compound was incomplete. By employing this model to collected data, assuming an initial percentage of sp^2^ (55%) and sp^3^ (20%) as reported by Ferrari et al. [27] and Ong et al. [28], we could estimate the loading time in oven at the above estimated temperature of 700 °C. Indeed, starting from the measured final percentage of sp^2^ (65%) and sp^3^ (10%), if one considers a volume of 1 mm^3^ of amorphous carbon, this transformation process involves about 1.5 × 10^19^ atoms in sp^3^ configuration (2.89 × 10^18^ transitions involving four atoms in tetrahedral coordination which change their hybridization to form a ring with sp^2^ coordination). Assuming an average transition time of ~10^−4^ s, it corresponds to a total time of

*t* = *τ*⋅(*n° of transitions*) = 10^−14^ s × 2.89 × 10^18^ = 2.89 × 10^4^ s (1)

As for the loading time, it means a permanence of about 8 h in oven at about 700 °C per mm^3^ of amorphous carbon content in the pottery, accordingly to the production technique in the late Early Bronze Age in the Jordan land.

In a further attempt to characterize ancient pottery, we combined Raman spectroscopy with computational chemistry simulation. In this case, we were concerned with the study of the drying oil technique used in Mesopotamian potteries from Kish (mid-third millennium B.C.), stored in the Ashmolean Museum of the University of Oxford (Oxford, UK) [29]. A portable Raman instrument, with excitation at 1064 nm was used also in this analysis, paired with microluminescence investigation (see details in the cited work). We performed Density Functional Theory (DFT) simulations by using clusters of atoms to model the structure of the molecules and their possible reactions (all the DFT calculations were performed with the GAUSSIAN09 code [30] at the B3LYP/6-31G(d) theory level). 

The aim was the investigation of the pottery technology adopted from that ancient community, especially in relation to the painting procedure. Apart from the use of hematite and gypsum as red and white pigments, respectively, the exploitation of a near-infrared laser excitation allowed us to reveal the presence of specific oil compounds applied at the pottery surface. From the experimental results it was deduced that the oil was applied all over the surface of the pottery as a primer before the painting decoration with the use of the dry oil technique. We detected residual traces of the oil thanks to the fingerprint vibration at 1870 cm^−1^ which is ascribed to the C–C stretching of the cyclopropenoid compound of some fatty acids. In particular, this feature can be assigned to the exploitation of oils derived from sterculiaceae and malvaceae plants, and its investigation with respect to other vibrational markers allowed us to infer about the starting oil content and its degradation during aging. 

**Figure 3:**
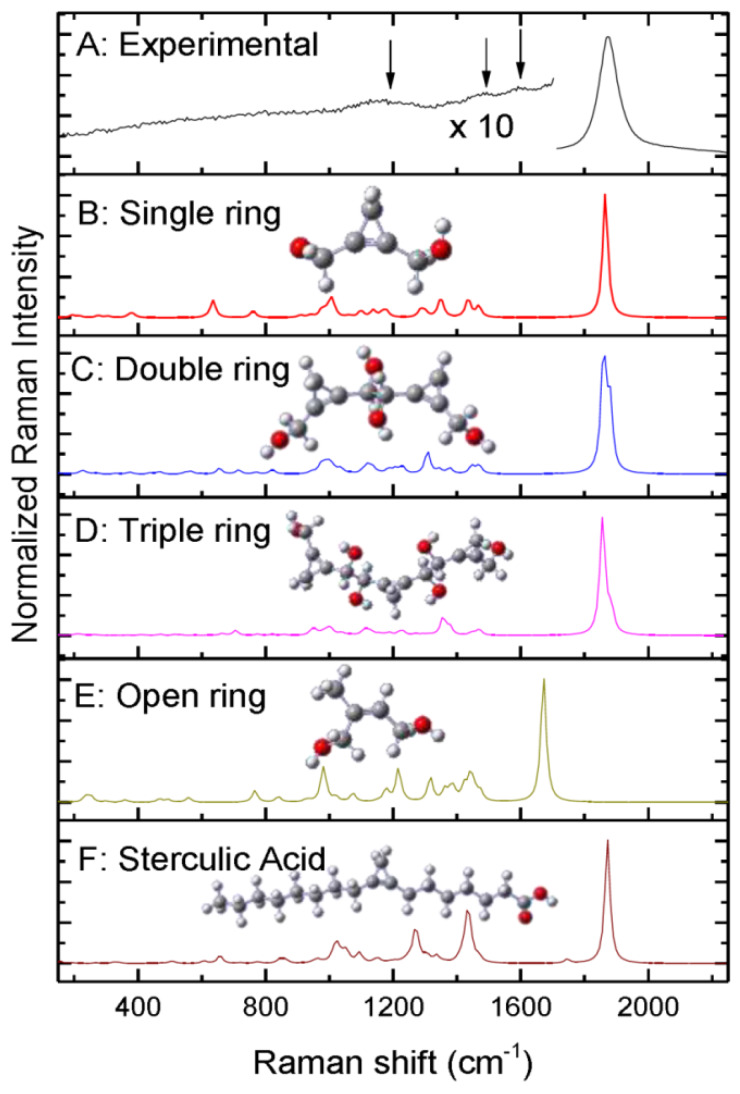
Comparison between experimental and simulated spectra of long chain and single ring of cyclopropenoid structure detected in Mesopotamian potteries from Kish (mid-third millennium B.C.). Cyclopropenoid compound was ascribed to some fatty acids derived from *sterculiaceae* and *malvaceae* plants. (Reproduced from work in [29] with the permission of Elsevier, copyright 2016.)

By using DFT calculations, as reported in Figure 3, we simulated the degradation process of the hypothesized initial oil and proposed the attribution of the final compound revealed by Raman analysis. 

Beside the relevance for the assessment of the technology applied by the art maker in that period, the appraisal of the cotton oil on that relics has a great historical value, suggesting the first commercial route and contacts with Arabic Gulf or Indian sub-continent cultures, where that specific cotton plants were cultivated.

A further exemplification on the combined use of Raman spectroscopy and models for dating purposes is represented by the paper of Kirmizi et al. [10]. In this work, a discussion about comparative dating or authentication of glazed pottery on the basis of Raman markers of protonic species included in the glazes is proposed. The incorporation is due to the corrosion processes as a function of time, chemical composition, and environmental conditions. The accumulation of protonic species such as water and hydroxyl groups on the surface of glassy silicates originates specific Raman features in the 2000 to 3800 cm^−1^ range. 

Raman measurements were acquired through a HR800 LabRam spectrometer (Horiba Scientific Jobin-Yvon) paired to a BX Olympus microscope equipped with different magnification objectives. Sampling data were collected by exciting the powders with the 457 nm laser line of an Ar+ ion laser (Coherent), keeping the laser power at ~6 mW.

The authors provided a comparison of all obtained data by plotting the so-called H_2_O/OH band area as an indicator of Raman intensity of the protonic species versus elapsed time since production date. The exploited model is the Arrhenius law for the diffusion of the protonic species in the glass:

(2) $$k=k_{0}e^{\frac{E_{a}}{k_{B}T}}$$

where the term *k* represents the rate constant and *k*_0_ and *E_a_* are the pre-exponential factor and the activation energy for this diffusion process, respectively. 

**Figure 4:**
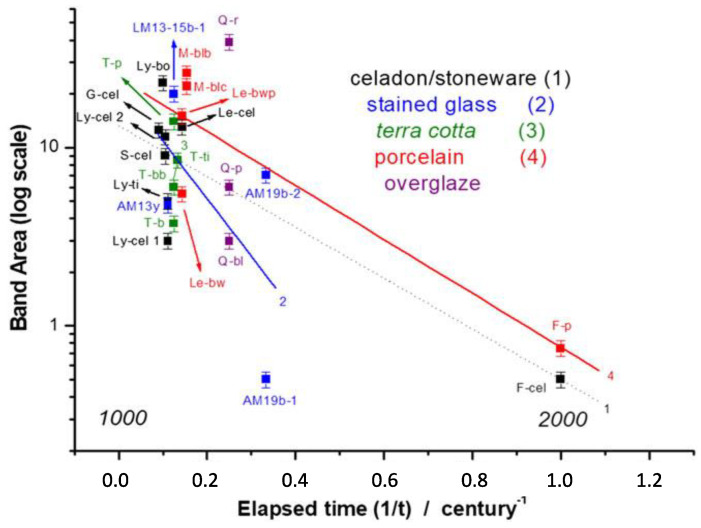
Arrhenius law applied to H_2_O/OH band area as a function of time passed since production date for celadons or stoneware, reference stained glasses, glazed terra cottas, porcelains, and porcelain overglazes. The groups are mapped with regression lines. (Reproduced from work in [10] with the permission of Wiley, copyright 2018.)

The results are reported in Figure 4 where Y scale is logarithmic and X scale is taken as inverse time, including the regression lines for the sample groups. The band area was mostly calculated in the 2300 to ~3300 cm^−1^ wavenumber range, with some small range variation to account for the shape of specific spectra.

As evidenced in Figure 4, regression lines are proposed in the semi-log plot to show the different time trend for the different groups, such as the celadons or stoneware, the reference stained glasses, the glazed terra cottas, porcelains, and porcelain overglazes, thus allowing the authors to achieve a dating model for the examined samples. 

### 2.2. Cellulose Materials

An important area of investigation in cultural heritage field where synergy between Raman spectroscopy and modeling find application is the one of cellulose materials. Ancient books and papers represent the historical memory of the human existence, our bequest from the past generations and the one we leave for the future. A long-standing problem concerns the degradation of the cellulose support of prestigious relics for conservation purposes. This problem was dealt with by many studies through the applications of diverse analysis techniques. In a recent work, we used a combined approach of Raman spectroscopy and mathematical modeling aimed to formulate a kinetic model allowing to enlighten the conservation state of ancient paper and provide dating information [11]. In this study, to investigate the degradation of cellulose, we paid attention to the breaking of glucose chain units. We collected Raman spectra of two different book collections, a certified public collection from Biblioteca Universitaria di Cagliari (C1 - well conserved) and a public/private one (C2 - degraded), covering a period of seven centuries (XV–XXI). Acquired results were compared to literature data on cellulose. The output was a successful kinetic model able to correlate an estimated aging time with the degradation process of cellulose. The chosen vibrational observables were the band at 1100 cm^−1^ assigned to C-O-C inter-monomers bond, whose relative intensity depends on the cellulose chain length, and the 900 cm^−1^ band, paired to the crystallinity grade of cellulose [11,31]. These two bands display an opposite trend and a shear stress like effect is suggested as the genesis of the cellulose chain length shortening. Thus, we monitored the intensity of the 1100 cm^−1^ band with respect to a reference band, the 1376 cm^−1^ CH vibration. Indeed, while the intensity of the former is a measure of the length of the cellulose polymer, the latter is related to the single glucose unit, and its intensity is not related to the cellulose polymerization degree. These assignations where also confirmed by DFT simulation of cellulose structure. To model the degradation of the cellulose, we assumed that the length of the polymer chain *l* at a fixed time, corresponding to the number of inter-monomer bonds at that time, is proportional to the *I*_1100_/*I*_1376_ ratio, according to the following expressions,

(3) $${\left(\frac{I_{1100}}{I_{1376}}\right)}_{t=0}\propto l_{0}\;\mathrm{and}\;{\left(\frac{I_{1100}}{I_{1376}}\right)}_{t}\propto l_{t}$$

at *t* = 0 and at the generic time *t*, respectively. 

Thus, by substituting *l_0_* and *l_t_* in the solution of a first-order kinetic model (Equation (4) in the following), under the assumption that the proportionality constant is the same for both the lengths, we can retrieve the degradation parameter *k* (assuming the reported print date of the book) or estimate the effective age of the analyzed paper samples if the *k* parameter is known (the *k* parameter accounts for conservation conditions, such as relative humidity, light exposure, and mean temperature):

(4) $$t=-k^{-1}\ln\left(\frac{l_{t}}{l_{0}}\right)=-k^{-1}\ln\left[\frac{a{\left(\frac{I_{1100}}{I_{1376}}\right)}_{t}}{a{\left(\frac{I_{1100}}{I_{1376}}\right)}_{0}}\right]=-k^{-1}\ln\left[\frac{{\left(\frac{I_{1100}}{I_{1376}}\right)}_{t}}{{\left(\frac{I_{1100}}{I_{1376}}\right)}_{0}}\right]$$

In addition, following the definition proposed in [11,31], we also considered the ratio *m/m_0_*, accounting for the number of C—O—C inter-monomers bonds when the degradation process started with respect to the starting number *m*_0_

(5) $$\frac{m}{m_{0}}=\frac{1}{2.77}\frac{\frac{I_{1100}^{m}}{I_{1376}^{n}}}{1.29}$$

This ratio allows estimation of the residual percentage of C–O–C inter-monomer bonds calculated for an ancient degraded paper, representing important information for conservation purposes. Indeed, the proposed kinetic model allows estimating from one side the aging time, from the other side the real conservation status of ancient paper.

**Figure 5:**
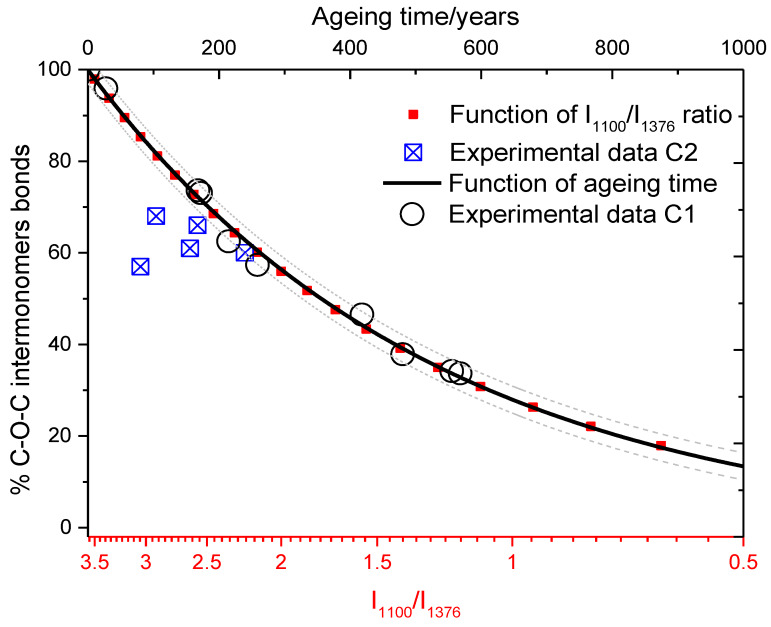
Aging and degradation model with experimental data: COC residual percentage of inter-monomer bonds as a function of aging time and *I*_1100_/*I*_1376_ intensities ratio. (Reproduced from work in [11] with the permission of Wiley, copyright 2018.)

The prediction of the model is reported in Figure 5, where the calculated residual percentage of C–O–C inter-monomer bonds is shown as a function of the ratio $\frac{I_{1100}}{I_{1376}}$, obtained as above illustrated, in the corresponding scale between t = 0 and t = 1000 aging years. The figure displays also some blue points derived from degraded paper, affected by foxing phenomenon and showing how this effect produces an accelerated aging of the reported samples. The estimated aging time was in very good agreement with the experimental data with 8% of accuracy.

As reported, starting from the observable $\frac{I_{1100}}{I_{1376}}$, derived from Raman spectra, the application of the kinetic model allowed to achieve a dating curve that can be successfully applied to ancient and modern paper when their conservation condition are controlled and well-known, or to show the effective age of a book affected by some degradation process.

### 2.3. Pigments 

Aging effect due to time and the interaction with the surrounding environment are very relevant also in the case of pigments, where it can drastically alter the perception of the colors as it was originally pursued by the painter. The case of the Van Gogh yellow in its famous sunflowers is probably the most renowned one [32,33]. Sometimes, the degradation can become so much pronounced it can ruin an artwork thus requiring large restorations, with all the ethical and technological problems related. 

Recently, we were involved in the study of calcium hydroxide used in art works joined with calcite as white pigment with the name of white lime or “Bianco di San Giovanni” and as binder in “a fresco” technique [34]. The term “Bianco di San Giovanni” finds origin into the book “II Libro dell’Arte”, where this name was first used for the calcium carbonated phase, mixed with calcium oxide/hydroxide [35]. To cite some relevant historical examples, this pigment was identified in walls and panel paintings of the Middle Edge, was applied in the 15th century to Gothic mural paintings, and it was also exploited by Giotto in many wall paintings, where it was mixed in the casein and egg tempera media. Besides its exploitation as a white pigment, calcium oxide/hydroxide is also largely used as ligand, because of its slow carbonation property (that is the slow formation of calcium carbonate, see below). Indeed, as mentioned before, calcium oxide/hydroxide find application also in “a fresco” technique as a ligand, where the mixture between pigment and water is employed to wet lime plaster.

The three compounds—calcium oxide, calcium hydroxide, and calcium carbonate—are the principal constituents of the carbonation/calcination cycle, which can be summarized as follows.

1. ${\mathrm{CaCO}}_{3}+\mathrm{heat}\to \mathrm{CaO}+{\mathrm{CO}}_{2}$
2. $\mathrm{CaO}+H_{2}O\;\to \mathrm{Ca}{\left(\mathrm{OH}\right)}_{2}$
3. $\mathrm{Ca}{\left(\mathrm{OH}\right)}_{2\;}+{\mathrm{CO}}_{2}\to {\mathrm{CaCO}}_{3}+H_{2}O$

In this case, the observable in the Raman spectrum consists in a broad double band positioned around 700–800 cm^−1^, typically observed when exciting at 1064 nm (the 700 to 800 cm^−1^ range corresponds to the infrared region around 1170 nm) [36]. The attribution of this band is still debated, with even its vibrational or luminescent character being questioned, but it is known that its observation and its spectral features depend on the environmental interaction, accounting also for time and temperature of the interaction process, mainly because of the carbonation/calcination cycle previously reported. For this reason, we decided to monitor the selected band under different experimental conditions and its relationship with the vibrational band at 1087 cm^−1^ associated to calcium carbonate (calcite). 

**Figure 6:**
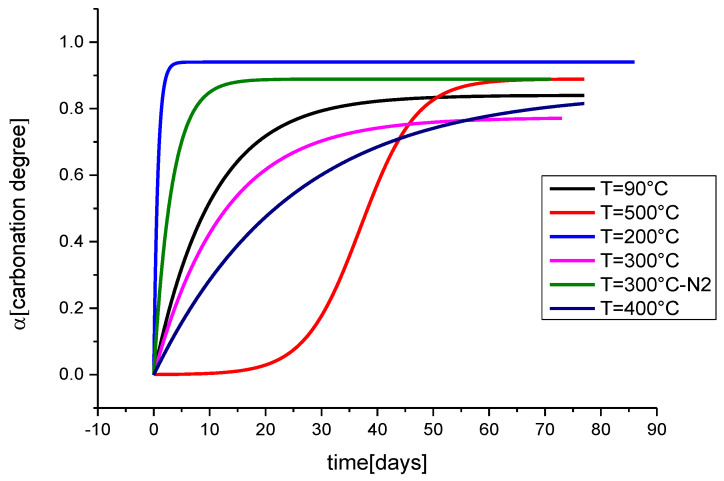
Kinetic model applied to experimental data using Equation (6). Samples of calcium hydroxide were treated at different temperature ranging from 90 °C to 400 °C.

This observable was studied in synthetic samples of calcium hydroxide, derived from the above cycle, as a function of temperature treatment (room temperature to 500 °C) and time. The nature of the band at 780 cm^−1^ was associated to structural luminescent defects due to the interaction with environmental CO_2_ or O_2_ and correlated with the formation of calcite band because of the carbonation process. Moreover, in this case, we proposed a kinetic model and, by assuming a first order kinetics, a rate equation was found:

(6) $$\alpha =\gamma \left[1-R_{n}\left(t\right)\right]=\gamma \left(1-e^{-\frac{t}{\tau }}\right)$$

where *α* is the degree of carbonation; *τ* indicates the characteristic time of the process; and *γ*, representing the asymptotic value in the graph of Figure 6, corresponds to the maximum carbonation relative value. 

The model considers the normalized ratio *R_n_* which represents the carbonated fraction of the compound with respect to the initial composition:

(7) $$R_{n}\left(t\right)=\frac{R_{t}}{R_{0}}=\frac{\frac{I_{780}}{I_{1087}}\left(t\right)}{\frac{I_{780}}{I_{1087}}\left(0\right)}$$

where the term $\frac{I_{780}}{I_{1087}}\left(0\right)$ corresponds to the relative initial percentage of emitting defective centers as compared to the amount of calcite crystals after the thermal treatment, and $\frac{I_{780}}{I_{1087}}\left(t\right)$ represents the relative amount of the defects at the time *t* of the ongoing carbonation process. The application of the proposed model to experimental data can be found in Figure 6 where the curves are plotted also as a function of the temperature. The curve at T = 500 °C presents a different trend (second-order kinetics) with respect to those obtained at lower temperature. This behavior suggests a second carbonation process due to direct conversion of calcium oxide to calcite. Actually, at T = 500 °C calcium hydroxide dissociates forming calcium oxide which starts a contemporary carbonation reaction [36]. 

As time and temperature are correlated, one can exploit the model to retrieve information on the thermal treatment undergone by the pigment or to trace its time trajectory. For example, the results obtained from thermal treatment can be used to recognize a possible thermal exposure of a mural painting or previous thermal treatment of the lime paste used to prepare the painting substrate. Alternatively, as the intensity of the bands is monitored as a function of the time, one can develop a dating model of the “a fresco” technique. 

Beside the aging concern, the analysis of the pigments is also relevant for authentication and tracing purposes, to unveil a specific signature of an artist or the painting technique adopted, find possible false attribution, or disclose commercial exchanges and diffusion of artworks and art technology [37]. When tracing and geographical trajectories are the target, the Raman spectroscopy can be usefully combined with multivariate analysis. Indeed, the combined application of Raman spectroscopy with multivariate analysis finds success in the field of pigments, especially when a mixture of components and ligands deserves to be studied in detail. An important example is given by Navas et al. [38] who proposed an articulate Principal Component Analysis (PCA) of a mixture (pigments and yolk or tempera) with the intent to discriminate the contribution of single components in producing the color effect. The work is based on the combination of first derivative processing of Raman spectra and a multivariate approach in order to discriminate the principal element of selected compounds. In particular, the authors focused the attention to different paint models presenting pure blue dyes (azurite, lapis lazuli, and smalt), pure red dyes (cinnabar, minium and raw Sienna), pure white dyes (lead white, chalk, and gypsum), and pure egg yolk as a ligand. The authors also examined tempera models consisting in a mixture of the above dyes with the ligand. Principal Component Analysis (PCA) was carried out distinctly on first derivative Raman features obtained from the pigment of the model samples (white, blue, or red). Raman spectra were collected by a Renishaw Invia Raman microscope system coupled to a Peltier-cooled CCD detector and a Leica DMLM microscope with mean spectral resolution of about 1 cm^−1^ in the 3800 to 200 cm^−1^ range. The laser excitation was provided by an Ar laser at 514.5 nm.

The authors built three data matrices, one for each color, including at first 30 spectra of tempera models, 30 ones of pure dyes, and 20 spectra of pure egg yolk ligand samples. Therefore, each color matrix was formed by 80 starting reference spectra. The PCA was carried out using both the covariance data matrices (scaling by mean-centered data) and the correlation data matrixes (scaling by unit variance). For specific details see the cited reference.

**Figure 7:**
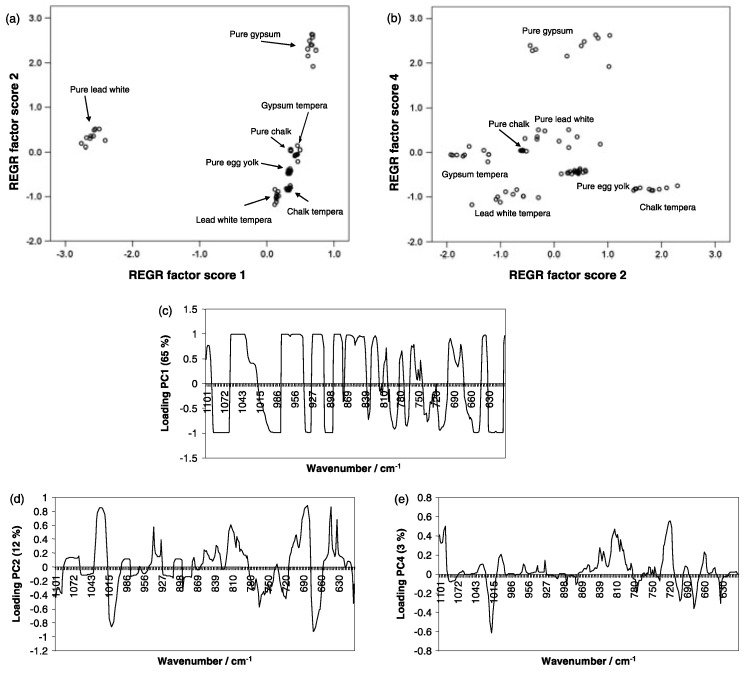
White model samples: (**a**) score plot of PC1 and PC2; (**b**) score plot of PC2 and PC4; (**c**) loading plot of PC1; (**d**) loading plot of PC2; (**e**) and loading plot of PC4. (Reproduced from work in [38] with the permission of Wiley, copyright 2010.)

Figure 7 plots the example of white model sample showing how score factors are correlated with principal components, allowing clustering of the information. 

As evidenced in the work proposed by Navas et al., three principal components, PC1, PC2, and PC4, were determinant to the discrimination procedure. In particular, the contribution of each of the three white pigments is observed in the PC1 loading plot (maxima and minima associated to all single reference compounds). The PC2 loading plot clearly indicates the contribution of the gypsum-laden samples (minima at 1007 cm^−1^ for example) and from the loading plot of PC4, the preeminent contribution of chalk is retrieved (see maximum at 1087, 811, and 717 cm^−1^). Through this approach, single contribution of reference pigments was determined and can be exploited for tracing and authenticity purposes.

### 2.4. Jewels and Gemstones

Raman spectroscopy, in combination with statistical approach, also finds application in ancient relics whose content reveals the presence of jewels or gemstones. Once again, the combined use of experimental data and model-oriented analysis is suitable for discriminating the authenticity and the provenance of materials. Smith, in [39], proposed a model to operate a semiquantitative chemical analysis of mineral solid solutions by multidimensional calibration of Raman wavenumber shifts and mathematical calculation by simultaneous equations. Raman spectra were collected by using a DILOR mobile Raman microscope spectrometer equipped with optical fibers and a green 532 nm laser. The model, following the method adopted by Smith, was proposed to investigate garnet hexary gemstones X_3_Y_2_Si_3_O_12_ forming a solid solution and aimed to discover how the end-members of the garnet can change the vibrational properties. Starting from a general formula of the hexary garnet group (Mg^2+^, Fe^2+^, Mn^2+^)_3_^xii^ (Al^3+^, Fe^3+^, Cr^3+^)_2_^vi^ (Si^4+^)3^iv^ O_12_, all the six equivalent garnets were found as a function of the end-member (pyrope, almandine, spessartine, grossular, andradite, and uvarovite).

The model was constructed by evaluating the band shift of the Raman spectra as a function of the mutual composition and the progressive phase variation. 

**Figure 8:**
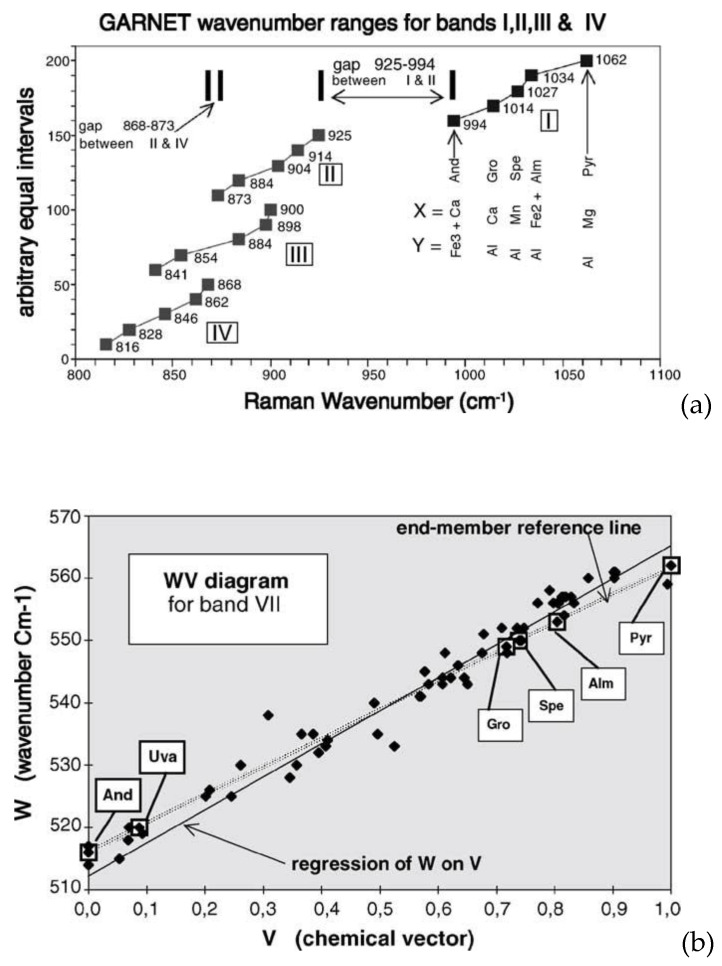
Experimental data determined by Smith et al. and the application of RAMANITA method: (**a**) plot of wavenumbers of bands I–IV for the garnet system Pyr–Alm–Spe–Gro–And–Uva; (**b**) the WV diagram for band VII in the Pyr–Alm–Spe–Gro–And–Uva garnet system. (Reproduced from [39] with the permission of Elsevier, copyright 2005).

Figure 8a shows the example of band shifting related to peaks I–IV for the end-members in the garnet system Pyr–Alm–Spe–Gro–Uva-And. The band overlapping prevents a facile solution based on relative contribution of individual components. Smith defined a “general multicomponent vector formula” able to express, for each analyzed *S_k_* sample, the contribution of each end-member to a specific *P_i_* band in the spectrum:

(8) $$V_{ik}=\left(X_{ak}\phi_{ak}\right)+\left(X_{bk}\phi_{bk}\right)+\left(X_{ck}\phi_{ck}\right)+\left(X_{dk}\phi_{dk}\right)+\left(X_{ek}\phi_{ek}\right)+\left(X_{fk}\phi_{fk}\right)$$

where *X_nk_* is the proportion of *n* end-member in sample *S_k_* and *Φ_nk_* is a normalizing factor related to each *n* end-member expressed as the ratio between the band shift values Δ*n* and the total range Δ*h* ($\phi_{nk}=\frac{\Delta n}{\Delta h}$*)*. Figure 8b plots the WV diagram for band VII showing all 40 garnets in the studied system providing the solution of the mutual composition.

Based on this vectorial model, many studies were able to successfully examine the graphical spread of single material whose contribution had different geographical provenance. The work proposed by Koleini et al. [40], for example, analyzed fourteen glass beads and one glass fragment from Khami-period (AD 1400–1830) sites of Danamombe, Naletale, Gomoremhiko, Nharire, and Zinjanja, in Harare, Zimbabwe. The intent was to assign the occupation domain of that land basing the analysis on jewels provenance. The results discriminate stratigraphic context assigning a period to each bead (from 10th–19th centuries) and relative provenance. 

The study, based on Raman measurements and a multivariate approach, took into account the relationship between Si—O bending and stretching modes present in the glass matrix of found beads. In fact, the position of mentioned band in the spectrum is a function of cations bonded to the glass matrix tetrahedron Si—O, when a pigment is applied to the bead forming a layer of ceramic glass. 

Raman spectra were obtained by using a T64000 micro-Raman spectrometer (HORIBA JobinYvon, Longjumeau, France). A krypton–argon mixed-gas laser was applied as wavelength source at 514.5 nm. 

**Figure 9:**
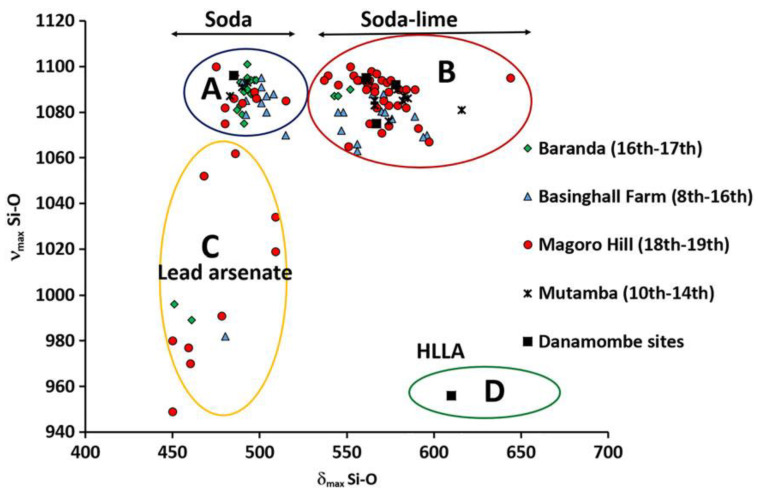
Representation of the relationship between the bending and stretching vibration bands of the SiO_4_ network. Points are classified in four groups with different composition. (Reproduced from work in [40] with the permission of Wiley, copyright 2019.)

Classification of glass from Raman spectra is achieved by reporting the large stretching vibration signal of SiO_4_ (in the 1000 cm^−1^ range) as a function of the bending peak maxima (in the 500 cm^−1^ range) as reported in Figure 9. This plot indicates the perturbation on the SiO_4_ tetrahedron exerted by adjacent ions (sodium, potassium, lead, and calcium) and the degree of polymerization. The classification was formerly applied to southern African beads recovered at Danamombe, Basinghall Farm, Mutamba, Magoro Hill and Baranda sites, producing four distinctive groups: Soda cluster (A), Soda-lime cluster (B), Lead arsenate cluster (C), and lime/potash cluster (D) having high lime and low alkali content (HLLA). 

In particular, group A is characterized by the presence of soda and high content of alumina (>7.9 wt %), suggesting a typical provenance form South Asia; group B, with high content of lime (7–11.8 wt %) and magnesia (>1.5 wt %), but lower percentage of alumina (3.3–6 wt %), is compatible with 10th century manufacture from South Europe (Italy, Spain and Portugal); group C evidences the use of lead arsenate pigment, typical European procedure starting from 18th century; group D having lime/potash composition (HLLA), is ascribed to European manufacture from post-medieval to the early 18th century period.

The multivariate analysis of Raman shifts of bending and stretching modes allowed the univocal assignment of glass relics to specific historical period and geographical sites, thanks to the relationship between structural vibrations and the presence of specific cations or pigments applied on the surface for decoration.

An analogous strategy was exploited by Peris-Dìaz et al. [41], where Raman spectroscopy, paired to partial least squares (PLS) model, was employed for classifying amber samples in agreement with their provenance and geological age. 

Raman measurements were executed by means of a Nicolet Magna 860 FT-IR spectrometer coupled with a FT-Raman tool. The samples were excited by 1064 nm laser light and the spectral data were collected at 23 °C in the 100 to 3800 cm^−1^ range with a spectral resolution of 4 cm^−1^. 

Raman spectra were recorded on samples from Czech Republic and Baltic region, and relics belonging to Upper Cretaceous and Cenozoic ages. As geographical and geological indicator the authors considered the intensity of the 1645 cm^−1^ and 1450 cm^−1^ bands, accounting for their ratio as a meaningful monitor. Indeed, the ratio of the intensities of the two bands allows estimating the geological age of the samples through the evaluation of the oxidation degree of the fossil resins, as related to the reduction of double bonds.

The study divided the amber on the basis of structural chemical features individuating two classes: succinite amber (Baltic) and valchovite amber (Moravian and Czech Republic). The classification was applied to 25 samples belonging to the collection of the Cultural Heritage Research Laboratory of the University of Wroclaw (Wroclaw, Poland), which gathered data from European, Asian, and American amber. 

**Figure 10:**
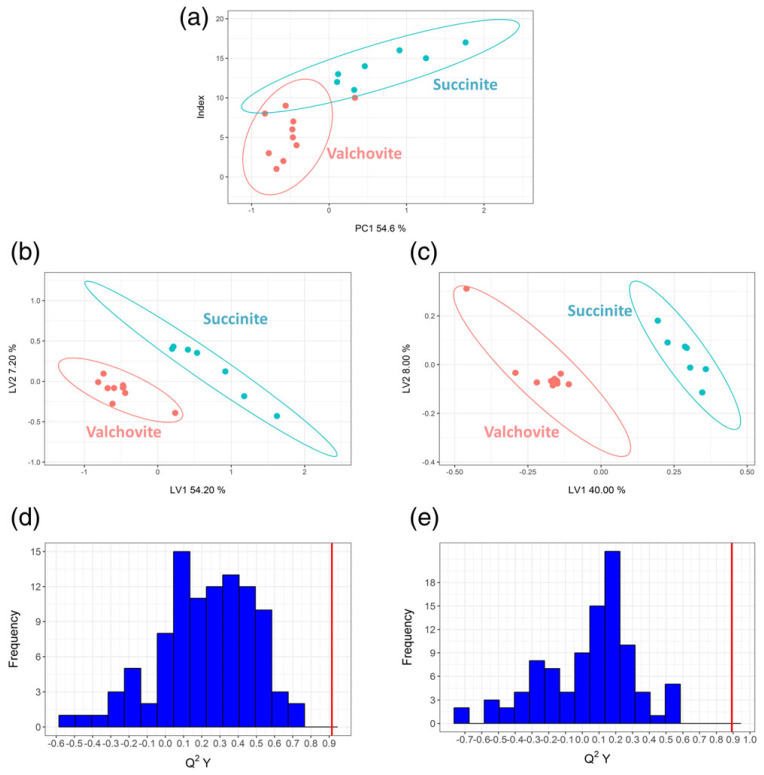
PCA-scores and PLS-scores plots of analyzed succinite and valchovite ambers (**a–c**). Panel (c) refers to the 1100 to 1800 cm^−1^ range. Density plot of the Q^2^Y values account for the full vibrational spectra (**d**) and the 1100 to 1800 cm^−1^ range (**e**). (Reproduced from work in [41] with the permission of Wiley, copyright 2017.)

Algorithm PLS (linear two-class locator) works with the variance–covariance matrices, derived from **X_ij_** matrix object-variable by means of multivariate method and scoring the values as a function of defined classes (+1, 0, −1). Cross-validation was employed to define the number of latent variables (LV) required for each interval using the Y explained variance (R^2^) and the predictable Y variation values (Q^2^Y), where Q represents the residuals (Figure 10, more details can be retrieved in the study of Peris-Dìaz). As shown in Figure 10, from the ratio *I*_1645_/*I*_1450_ the authors constructed the PCA-scores and PLS-scores diagram obtaining a perfect classification between succinite and valchovite, correlating them, in a second step, to Upper Cretaceous or Cenozoic ages. 

Once again, the combined use of Raman fine analysis and models based on multivariate approach provided important results about provenance and dating purposes of ancient samples. 

### 2.5. Other Possible Cases

The proposed applications of combined use of Raman spectroscopy and mathematical model can be extended to infinite cases in which analyzable trends are found. In the field of cultural heritage, for example, other possible cases can have significance for the study of degradation processes connected to parchment aging. The breakage of important bonds in the parchment structure, as a function of the time, would be easily detected by Raman spectroscopy [37,42,43]. Another example can be found in the progressive acidification and degradation of medieval black inks (principally iron gall), which causes important problems in manuscript corrosion [44,45]. Moreover, in this case, the progressive acidification of the ink can be monitored by means of Raman spectroscopy and a kinetic model can be proposed to study the conservation state. In order to investigate the above-mentioned problems, further studies are currently underway.

## 3. Conclusions

In the last decades, Raman spectroscopy became an efficient non-destructive technique for investigations in the field of cultural heritage. Raman spectroscopy showed its validity not only as an efficient tool for compositional analyses or as a useful technique for imaging procedure, besides very recent applications in non-destructive stratigraphy investigations, but it also plays a key role if combined with a mathematical model. In fact, as reported in this work, in many cases this technique deepened the study of artworks with the help of supporting models able to connect environmental variables with the aging time or provenance, thus allowing to trace time and geographical trajectories of the investigated relics. As demonstrated in the reviewed illustrative example studies, the application of this combined elements found results in the case of potteries, ancient and modern paper, pigments degradation, gemstones, and ancient jewelry, and can be potentially employed in many diverse cases. The aim of the review is to promote this combined approach showing that Raman spectroscopy can be very powerful and innovative especially in the field of cultural heritage for conservation and restoration purposes.
